# Nanoscale size effects in crystallization of metallic glass nanorods

**DOI:** 10.1038/ncomms9157

**Published:** 2015-09-01

**Authors:** Sungwoo Sohn, Yeonwoong Jung, Yujun Xie, Chinedum Osuji, Jan Schroers, Judy J. Cha

**Affiliations:** 1 Department of Mechanical Engineering and Materials Science, Yale University, New Haven, Connecticut 06511, USA; 2 Energy Sciences Institute, Yale West Campus, West Haven, Connecticut 06516, USA; 3 Department of Chemical & Environmental Engineering, Yale University, New Haven, Connecticut 06511, USA

## Abstract

Atomistic understanding of crystallization in solids is incomplete due to the lack of appropriate materials and direct experimental tools. Metallic glasses possess simple metallic bonds and slow crystallization kinetics, making them suitable to study crystallization. Here, we investigate crystallization of metallic glass-forming liquids by *in-situ* heating metallic glass nanorods inside a transmission electron microscope. We unveil that the crystallization kinetics is affected by the nanorod diameter. With decreasing diameters, crystallization temperature decreases initially, exhibiting a minimum at a certain diameter, and then rapidly increases below that. This unusual crystallization kinetics is a consequence of multiple competing factors: increase in apparent viscosity, reduced nucleation probability and enhanced heterogeneous nucleation. The first two are verified by slowed grain growth and scatter in crystallization temperature with decreasing diameters. Our findings provide insight into relevant length scales in crystallization of supercooled metallic glasses, thus offering accurate processing conditions for predictable metallic glass nanomolding.

## Abstract (web summary)

It is crucial to avoid crystallization in metallic glasses, which show appealing mechanical properties associated with their amorphous structures. Sohn *et al*. identify unusual nucleation kinetics in metallic glass nanorods, which exhibits a crystallization temperature minimum at a certain diameter.

## Graphical abstract

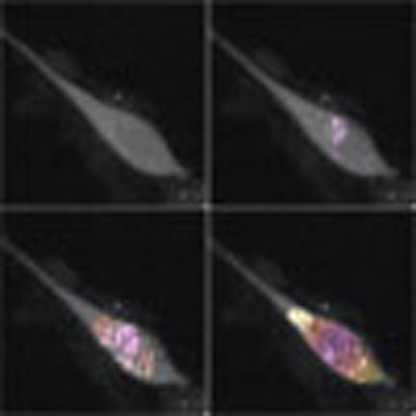

Nanoscale metallic glasses (MGs) offer novel technological applications^1^ due to their superior mechanical properties such as high mechanical strength, high elasticity, high fracture toughness and high corrosion resistance^2^,^3^,^4^,^5^,^6^,^7^,^8^,^9^. In some cases, their unique chemical compositions are favourable for catalysis^10^, bioimplants^11^ and antibacterial applications^12^. Owing to the absence of an intrinsic length scale, hierarchical structures with multiscale surface features from centimeters down to nanometers are possible for MGs to engineer novel material properties^10^,^13^, which is challenging in crystalline metals. Patterning of MGs is typically achieved by thermoplastic forming^14^—pressing heated MGs into molds, where processing temperatures are in the supercooled liquid region bounded by the glass transition temperature and crystallization temperature. Most readily, molding is realized at the highest temperature where crystallization can still be avoided, thus retaining the attractive properties of the amorphous material^15^,^16^. For reliable thermoplastic processing of multiscale structuring of MGs, crystallization kinetics at all length scales must be understood. On the macroscale, crystallization kinetics have been determined for a wide range of alloys, enabling predictable molding^15^. By contrast, the crystallization behaviour of MG-forming liquids at nanoscale dimensions has not been carefully considered. This is in part due to the non-trivial experimental difficulty associated with detecting and accurately recording the heat signals from nanoscale samples as they undergo crystallization in the differential scanning calorimetry experiments that are typically used, to good effect, for macroscale samples. Despite experiments on a large ensemble of nanoscale samples indicating accelerated crystallization^17^, it is unclear whether crystallization kinetics remains unaltered at nanoscale. Up to now, macroscale processing parameters have been used for nanoscale molding of MGs with often-unpredictable crystallization behaviours^18^. Recently, nanocalorimetry has been used to study nanoscale samples^19^,^20^. One major challenge of nanocalorimetry is accuracy, and calorimetry alone cannot directly relate heat release with nanoscale MG morphology. In contrast, *in-situ* transmission electron microscope (TEM) techniques can simultaneously introduce and visualize structural changes to reveal phenomena that cannot be observed from *ex-situ*, ensemble techniques. *In-situ* TEM has been used to observe the dynamic structural changes of nanomaterials, such as growth of nanocrystals^21^ and nanowires^22^, and phase transformations in glassy materials^23^,^24^, providing a near atomistic understanding of their underlying mechanisms.

Here, we apply *in-situ* heating of MG nanorods inside a TEM and visualize their crystallization characteristics at unprecedentedly small length scales. We directly observe the structural changes of MG nanorods during crystallization and reveal unusual intrinsic size effects on their crystallization temperature, marking a departure from continuous flow description of MG kinetics.

## Results

### *In-situ* TEM heating

**Figure 1:**
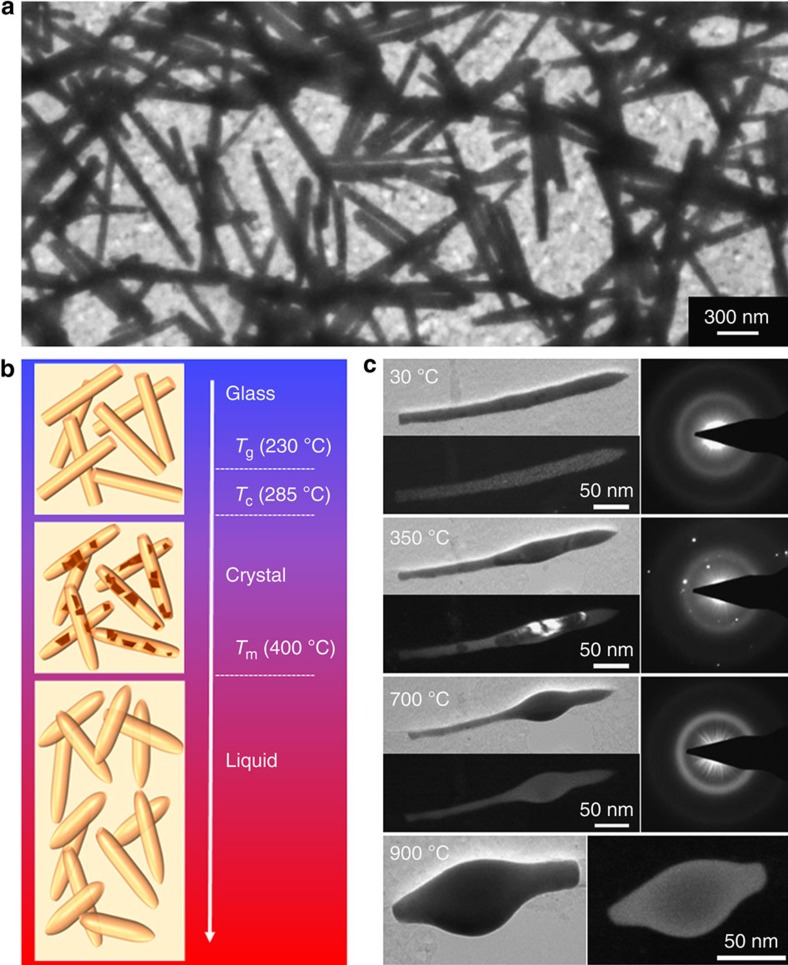
Phase transition of MG nanorods under *in-situ* TEM. (**a**) TEM image of MG nanorods pressed into a nanomold with 55-nm-diameter holes. They are drop casts on a thin ceramic film, which gets heated inside TEM. (**b**) Schematics for the morphological change of MG nanorods at different stages of *in-situ* heating. As-pressed nanorods exhibit uniform TEM image contrast (first panel). As temperature increases above crystallization temperature, *T*_c_, crystalline grains start to grow in MG nanorods (dark brown in the second panel). The grains are identified by TEM imaging or electron diffraction. As temperature increases above the melting temperature, *T*_m_, the crystallized nanorods turn to liquid phase (third panel). (**c**) TEM characterizations of a nanorod under *in-situ* heating. At 30 °C, the nanorod is amorphous, confirmed by the uniform TEM image contrast (top left: BF image, bottom left: DF image) and a diffusive ring pattern in SAED (right panel). Above *T*_c_ at 350 °C the nanorod is largely crystallized, reflected in contrast changes in the TEM images. The crystalline phase appears dark for BF (top left) and bright for DF (bottom left) TEM images, respectively. The corresponding SAED displays diffraction spots, indicating the presence of crystalline grains (right panel). At 700 °C, which is above *T*_m_, uniform TEM image contrast and diffusive SAED ring similar to those at 30 °C are observed to indicate that the nanorod is back to disordered state. As heating progresses with longer time, the nanorod becomes significantly deformed. At 900 °C, the nanorod becomes greatly shortened and balled-up.

Amorphous MG nanorods of Pt_57.5_Cu_14.7_Ni_5.3_P_22.5_ are prepared by a nanomolding technique^14^ (Supplementary Fig. 1). Figure 1a shows a representative TEM image of as-pressed MG nanorods on an *in-situ* TEM thermal grid that can be heated up to 1100 °C. The nanorods are heated from room temperature to above their liquidus temperature inside the TEM, and their structural changes during heating are observed using bright-field (BF) imaging, dark-field (DF) imaging and selective area electron diffraction (SAED) (Fig. 1b,c). The glass transition (*T*_g_), crystallization (*T*_c_) and melting (*T*_m_) temperatures of bulk samples are indicated in Fig. 1b.

### Size-dependent crystallization temperature

**Figure 2:**
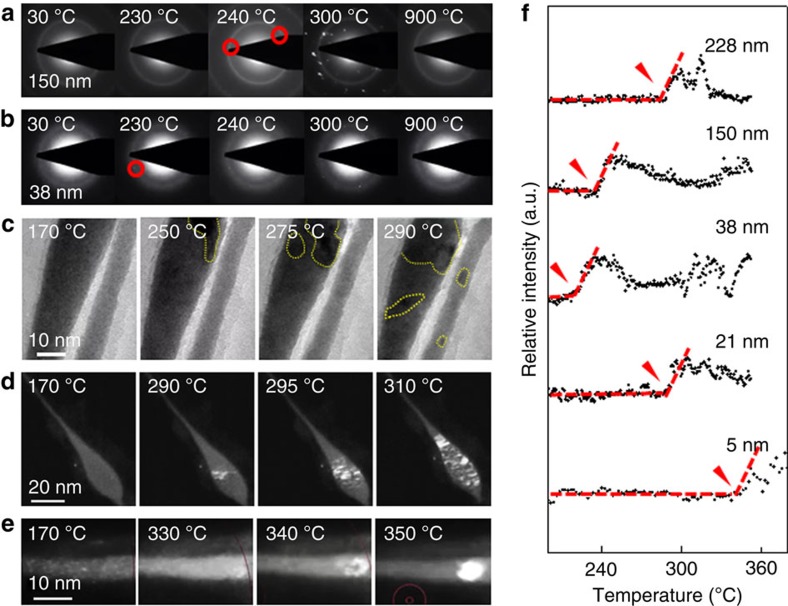
*In-situ* characterization of the *T*_c_ of nanorods with varying diameters. (**a**–**e**) Individual nanorods were heated inside the TEM at a constant ramping rate of 0.67 °C s^−1^ and their crystallization kinetics were monitored in real time. SAED and TEM imaging are used to identify the crystallization kinetics and *T*_c_. For thicker nanorods (diameter >∼35 nm), *T*_c_ was determined by identifying the onset of the first SAED spot. For smaller nanorods (diameter <∼35 nm), *T*_c_ was determined by identifying the TEM image contrast change in BF and DF modes. (**a**,**b**) Snapshot SAED to compare the crystallization kinetics of nanorods of 150 and 38 nm diameters, respectively. The red circles indicate the onset of the first diffraction spot, reflecting the onset of crystallization. (**c**) Snapshot BF TEM images to compare the crystallization kinetics of nanorods of diameters <25 nm. The dotted yellow lines indicate crystalline grains, which form at a lower temperature for the thicker nanorod. (**d**) Snapshot DF TEM images to show the crystallization kinetics of a tapered nanorod, revealing that the formation of crystalline grains (bright contrast) initiates on the thicker region. (**e**) Snapshot DF TEM images to show the crystallization kinetics of an extremely thin, ∼6-nm-diameter nanorod, showing that crystalline grains form at ∼340 °C, which is over 50 °C higher than the *T*_c_ of bulk MGs. (**f**) Quantification of *T*_c_'s for nanorods of various diameters based on the intensity change of TEM image contrasts. The abrupt increase of the relative intensity in each trace (denoted by red arrows) reflects the advent of first crystalline grains, which is defined as *T*_c_ (see Methods). The decrease of the intensity after *T*_c_ is attributed to the dynamic motion of grains under heating, so initial diffraction spots do not last.

We characterize the crystallization behaviour of individual nanorods in the diameter range of ∼6–330 nm under *in-situ* heating. *T*_c_ is detected from SAED or DF TEM movies during heating (Fig. 2) as the onset of a rapid increase in the electron intensity in the back focal plane (SAED) or image plane (DF), respectively. SAED *in-situ* movies reveal that a thinner nanorod crystallizes at a lower *T*_c_ than a thicker rod (Fig. 2a,b, Supplementary Movies 1 and 2). The general trend is a monotonically decreasing *T*_c_ with decreasing nanorod diameter down to ∼25 nm. For nanorods below 25 nm, *T*_c_ shows a reverse trend, increasing rapidly with decreasing nanorod diameter. Figure 2c shows snapshot BF TEM images that compare the crystallization kinetics of two nanorods with diameters below ∼25 nm. Here, a thinner nanorod crystallizes at a higher *T*_c_ than a thicker one, which is the opposite behaviour of the ones observed in Fig. 2a,b. This reversed trend of *T*_c_ is best visible in tapered nanorods with narrowing diameters. Figure 2d shows snapshot DF TEM images of a tapered nanorod under *in-situ* heating (Supplementary Movie 3). Crystalline grains grow first in the thicker region and subsequently grow towards the thinner region. Remarkably, the thinner region, which is below 5 nm, never crystallizes. Crystallization of an ∼6-nm nanorod occurs at ∼340 °C (Fig. 2e), which is ∼100 °C higher than the *T*_c_ for a 150-nm nanorod (Fig. 2a). Figure 2f presents quantitatively measured *T*_c_'s of nanorods of various diameters under *in-situ* heating (see Methods). It reveals the initial decrease of *T*_c_ with decreasing nanorod diameter and a rapid increase of *T*_c_ below 25 nm. No detectable changes in the chemical composition of nanorods were observed during *in-situ* heating by energy-dispersive X-ray spectroscopy (Supplementary Fig. 2) and cyclic heating experiments (Supplementary Fig. 3), ruling out chemical and electron beam effects as the origin for the observed *T*_c_ change.

**Figure 3:**
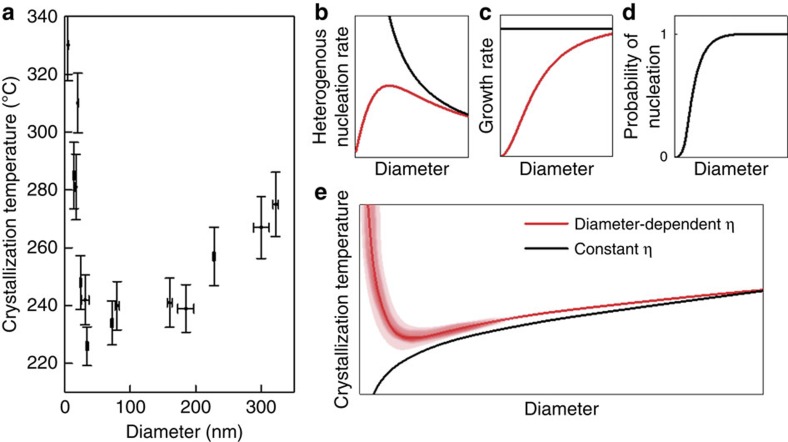
Diameter-dependent crystallization kinetics of MG nanorods. (**a**) Correlation of *T*_c_ vs. nanorod diameter. All data were collected from non-tapered nanorods of uniform diameters. The uncertainty in *T*_c_ is calculated from the standard deviation of the temperature fluctuations during heating. (**b-d**) Schematics to illustrate the effects of surface-induced heterogeneous nucleation (**b**), apparent viscosity (**c**) and reduced nucleation probability (**d**) on crystallization kinetics. Red curves show the combined effect of the size-dependent surface-induced heterogeneous nucleation and apparent viscosity. The combined effect results in a size-dependent transition of the heterogeneous nucleation rate, while the growth rate monotonically decreases with decreasing diameter. This is due to the fact that diffusivity and the jumping frequency are inversely proportional to the viscosity. Black curves in **b** and **c** consider only the surface-enhanced nucleation. (**e**) The combined effect of size-dependent nucleation and growth rates results in a minimum of *T*_c_ at a certain diameter. The shaded area in red indicates the scatter in *T*_c_'s, reflecting the stochastic nature of nucleation at the reduced nucleation probability.

A summary of *T*_c_'s for all considered nanorods is given in Fig. 3a. *T*_c_ monotonically decreases with decreasing diameter down to ∼25 nm, below which it rapidly increases. This non-monotonic crystallization behaviour is discussed in the context of size-dependent apparent viscosity, nucleation probability and heterogeneous nucleation, all of which affect nucleation, growth rates and crystallization kinetics (Fig. 3b–e). For nanorods, heterogeneous nucleation on the surface of nanorods dominates over homogeneous nucleation, which would accelerate crystallization kinetics with decreasing nanorod diameter, thus lowering the *T*_c_ (Fig. 3b, black curve)^25^. Below a critical nanorod diameter, however, two additional factors affect the crystallization kinetics: reduced probability of nucleation and increase in the apparent viscosity.

## Discussion

**Figure 4:**
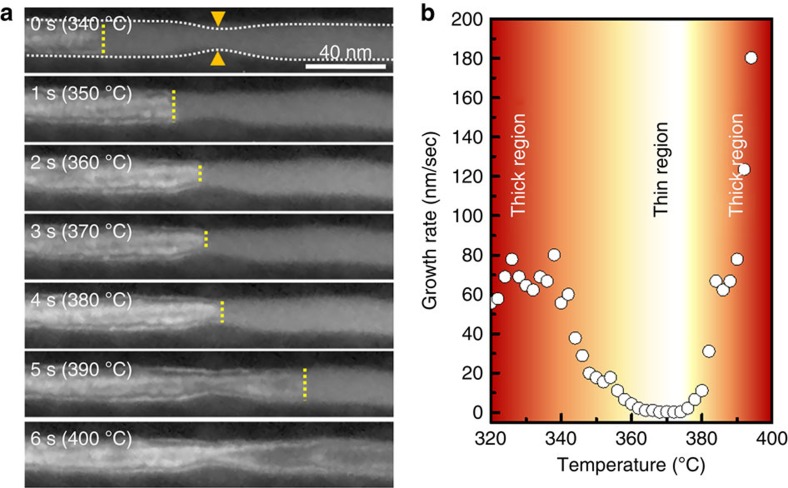
Direct verification of growth rate difference in a nanorod. (**a**) Snapshot DF TEM images to observe the growth kinetics in a nanorod of ∼17 nm with a narrow region (∼9 nm, denoted by orange arrows) in diameter. The dotted line in the top image outlines the nanorod morphology. The grain growth rate is estimated by measuring the lateral expansion of the crystalline grain front, reflected as the change of imaging contrast (yellow dashed lines). Note that the growth front passes through the neck at ∼370 °C. (**b**) Quantification of the crystalline grain growth rate. The grain growth rate is suppressed at the narrow region of the nanorod during crystallization. The growth rate for the thicker region is ∼65 and ∼180 nm s^−1^ at 330 and 394 °C, respectively, and the rate for the thinner region is 4.5, 0.3 and 11 nm s^−1^, at 360, 370 and 380 °C, respectively. The detailed experimental condition is described in Supplementary Note 2.

We discuss the increase in the apparent viscosity first. When the sample size approaches the size scale of intrinsic flow units such that the shear transformation zone can no longer freely operate, the flow resistance increases, causing apparent viscosity, *η*, to increase^26^. Such departure from a continuum flow description, reflected in the increase of viscosity with decreasing confinement sizes, or equally, increasing Knudsen number, has been observed in other viscoelastic systems, including polymers or glassy materials^26^. The increase in viscosity would lead to decrease in nucleation rate and grain growth rate, as the jumping frequency and the diffusion constant are inversely proportional to the viscosity (Fig. 3b,c, red curves)^27^. We have experimentally investigated the growth kinetics in a nanorod with a locally narrowed region (Fig. 4). Indeed, we observe a suppressed grain growth rate in the narrower region during *in-situ* crystallization (Supplementary Movie 4). The growth front significantly slows down through the narrow region, as indicated by the snapshot DF TEM images (Fig. 4). The growth mode appears to remain the same throughout the entire growth time. Thus, this is a direct consequence of the increase in the apparent viscosity, which leads to the slowdown of crystallization kinetics and the increase of *T*_c_, in agreement with our observation. Systematic size-dependent grain growth rate data are shown in Supplementary Fig. 4. Discussion on the growth mechanism can be found in Supplementary (Supplementary Figs 4,5).

**Figure 5:**
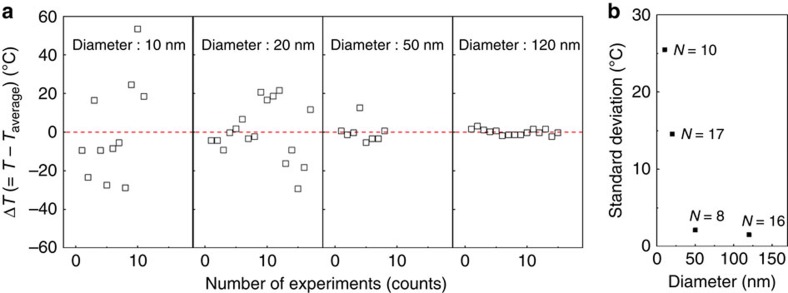
Statistical studies on *T*_c_'s as a function of nanorod diameter. (**a**) The measured temperature scatter (Δ*T*=*T*−*T*_average_), which is the difference between the crystallization temperatures (*T*_c_) and the average *T*_c_ (*T*_average_), and (**b**) the corrected sample standard deviation of nanorods with a diameter of 10, 20, 50 and 120 nm, obtained from *N* number of nanorods. The scatter increases with decreasing nanorod diameter, suggesting that the reduced probability of nucleation plays a critical role.

In addition to the increasing apparent viscosity and heterogeneous nucleation, reduced probability of nucleation critically affects the crystallization kinetics, particularly for small nanorods. As the number of nuclei scales with the surface area of the nanorod, the probability of nucleation is anticipated to decrease if the nanorod size approaches the mean distance between nuclei (Fig. 3d, Supplementary Fig. 6). This ultimately slows down the crystallization kinetics, reflected as increase in *T*_c_. Due to the stochastic nature of nucleation, the reduced probability of nucleation will result in a larger scatter in *T*_c_ for smaller rods. To confirm this, we performed *in-situ* crystallization experiments repeatedly and measured the scatter in *T*_c_ as a function of nanorod diameter. In agreement with our prediction, the scatter in *T*_c_ gradually increases for smaller nanorods (Fig. 5), pointing to the effect of the reduced probability of nucleation for small rods. The non-monotonic behaviour of *T*_c_ shown in Fig. 3a, the main finding of this work, still holds even in the presence of the scatter. We note that a decrease in melting temperature (*T*_m_), expected from the Gibbs–Thomson effect, does not alter the observed diameter-dependent *T*_c_ (Supplementary Fig. 7).

Size-dependent *T*_c_ that we map out for nanoscale MGs down to ∼5 nm lays the foundation for their stable processing. The increase of *T*_c_, owing to the reduced probability of nucleation, points to a larger processing window than previously assumed for feature sizes smaller than ∼30 nm to circumvent crystallization. By employing higher processing temperatures, current size limits for nanoscale MGs may be overcome. Fundamentally, our *in-situ* study of size effects on the crystallization behaviour of MGs reveals relevant length scales and a departure from a continuous flow description of MGs at the nanoscale.

## Methods

### Preparation of MG nanorods

The synthesis of Pt_57.5_Cu_14.7_Ni_5.3_P_22.5_ MG nanorods used in this study is described in detail in our previous paper^14^. Briefly, alloying of high-purity constituents with nominal compositions was carried out in a vacuum-sealed quartz tube using induction melting. Subsequently, the master alloys were fluxed with boron trioxide (B_2_O_3_) and vitrified by water quenching from a temperature of 1100 °C, which is 350 °C above the thermodynamic liquidus temperature. A commercially available porous anodized aluminium oxide (AAO) mold (Synkera Inc.) was placed between two custom-built heating plates, at a constant temperature of 260 °C (Supplementary Fig. 1a). A piece of bulk MG was placed and equilibrated on the AAO mold at 260 °C for 30 s (Supplementary Fig. 1b). Nanorods were thermoplastically formed by pressing the bulk MG into the selected AAO mold, under a load at a linear ramping from 0 to 100 kN (Supplementary Fig. 1c). The pressed sample was subsequently released from the mold by dissolving the AAO mold in a 20 wt.% potassium hydroxide (KOH) solution, heated at 80 °C for ∼10 h (Supplementary Fig. 1d). The released MG plate with nanorods attached was rinsed with distilled water and isopropyl alcohol (IPA). The rinsing process was repeated more than four times to minimize the residual salts and AAO mold fragments. Then, the nanorods were released and collected by sonication in IPA (Supplementary Fig. 1e). The minimum diameter of the AAO molds used was 13 nm. Nanorods of diameters <∼10 nm were stochastically produced in response to the dewetting process, by rapidly melting/quenching as-pressed nanorods inside the TEM column (∼10^−5^ Pa vacuum level). The melt/quench was carried out at 900 °C with a fast quenching rate of ∼10^6^ K s^−1^. Energy-dispersive X-ray spectroscopy characterizations (Supplementary Fig. 2) show no detectable differences in the chemical composition of nanorods irrespective of diameters and *in-situ* heating processes.

### *In-situ* TEM heating experiments

All TEM experiments were performed with a FEI Tecnai Osiris 200 kV TEM. As-pressed nanorods dispersed in IPA were transferred to *in-situ* TEM substrates, via a drop casting method. We used commercially available *in-situ* TEM heating grids (E-AHA) and a heating holder (Aduro 300DT System) manufactured by Protochips, Inc. The heating substrate contains a resistive ceramic membrane contacted with metal electrodes, which introduce Joule heating to the membrane. The ceramic membrane contains an array of holes, over which a thin carbon film is overlaid. Nanorods sitting on the amorphous carbon film were inspected for *T*_c_ identification, so there is no contribution of the ceramic on diffraction spots or TEM imaging. The temperature profile of each substrate was pre-calibrated by the manufacturer. For the identification of *T*_c_, nanorods were first rapidly heated to 170 °C (below *T*_g_) within 0.1 s. Subsequently, a slower heating with a ramp rate of 0.67 °C s^−1^ was applied from 170 to 350 °C. During the heating, nanorods were monitored in real time under active DF/BF TEM imaging modes or SAED modes, which were simultaneously video-recorded (Snagit software).

### Quantification of *T*_c_ based on the intensity change in TEM imaging contrast

Quantification of *T*_c_ for nanorods with varying diameters was carried out by a series of intensity profile measurements made over an image contrast variation. Image frames of the BF or DF TEM movies recorded during *in-situ* heating were aligned using an automated pixel-based plug-in application, TurboReg (Biomedical Imaging Group, Swiss Federal Institute of Technology, Lausanne, Switzerland)^28^ in ImageJ software (National Institutes of Health, Bethesda, MD)^29^. After alignment, two regions of interest (ROIs) were selected: one on the bright region of the sample (for BF/DF image movies) or on the first diffraction ring (for SAED pattern movies) and the other on the background. Intensity counts from the two ROIs were extracted from all image frames as a function of imaging time. The relative intensity profile for each nanorod was calculated as,


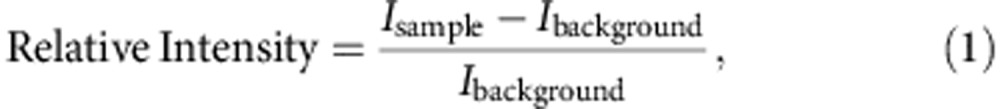


where *I*_sample(background)_ is the intensity of ROI from the sample (background). At the onset of crystallization, the relative intensity increases rapidly (Fig. 2f). We define *T*_c_ at the intercept of the constant background and the linear fit to the increasing relative intensity.

## Additional information

**How to cite this article**: Sohn, S. *et al*. Nanoscale Size Effects in Crystallization of Metallic Glass Nanorods. *Nat. Commun.* 6:8157 doi: 10.1038/ncomms9157 (2015).

## Supplementary Material

Supplementary Information Supplementary Figures 1-7, Supplementary Notes 1-7 and Supplementary References

Supplementary Movie 1 *In-situ* electron diffraction movie of a 150 nm-diameter nanorod.

Supplementary Movie 2 *In-situ* electron diffraction movie of a 38 nm-diameter nanorod.

Supplementary Movie 3 *In-situ* dark field image movie of a tapered nanorod (3-20 nm).

Supplementary Movie 4 *In-situ* dark field image movie of a 17 nm-diameter nanorod with a narrow region (~9 nm).
