# Supplementary material for: Nanoscale size effects in crystallization of metallic glass nanorods
Source: Nat Commun. 2015 Sep 1;6:8157. doi: 10.1038/ncomms9157 (PMC4569721; doi:10.1038/ncomms9157)
Supplement: Supplementary Information — Supplementary Figures 1-7, Supplementary Notes 1-7 and Supplementary References [file ncomms9157-s1.pdf]

Supplementary Figures

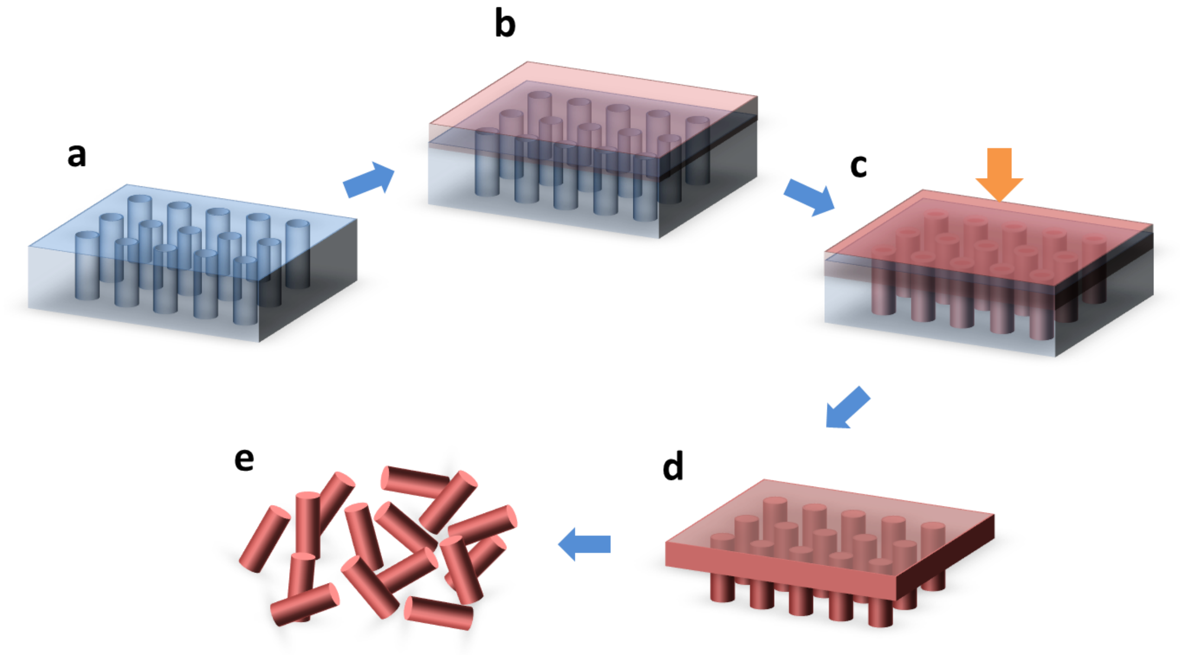

Supplementary Figure 1. Schematics to illustrate the nanomolding process. **a,** Anodized aluminum oxide (AAO) template is used as a mold for nanorods. **b,** Pt-based bulk MG is placed on the AAO mold and heated to the processing temperature 260 ^{o}C. **c,** The MG is thermoplastically formed into the mold under a linear applied force. **d,** The pressed sample is released by dissolving the AAO mold in a potassium hydroxide (KOH) solution. **e,** The nanorods are collected by sonication in IPA.

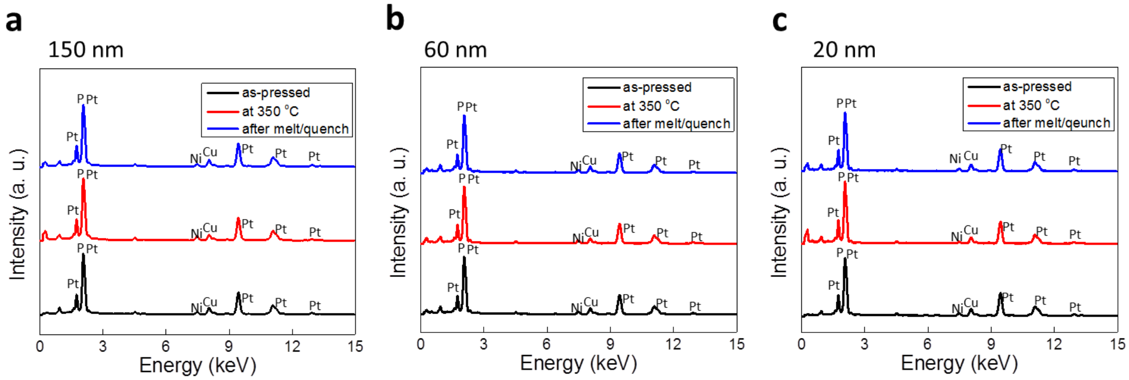

Supplementary Figure 2. Energy dispersive X-ray spectroscopy (EDX) characterizations to confirm the uniformity of chemical composition, irrespective of nanorod diameters and *in-situ* heating conditions. For the nanorods of different diameters (**a,** 150 nm, **b,** 60 nm, **c,** 20 nm), each EDX profile plot presents different *in-situ* heating conditions. Black, red, and blue represent the EDX profile for nanorods before heating, during heating at 350 ^{o}C, and after rapid melt/quench at 900 ^{o}C, respectively.

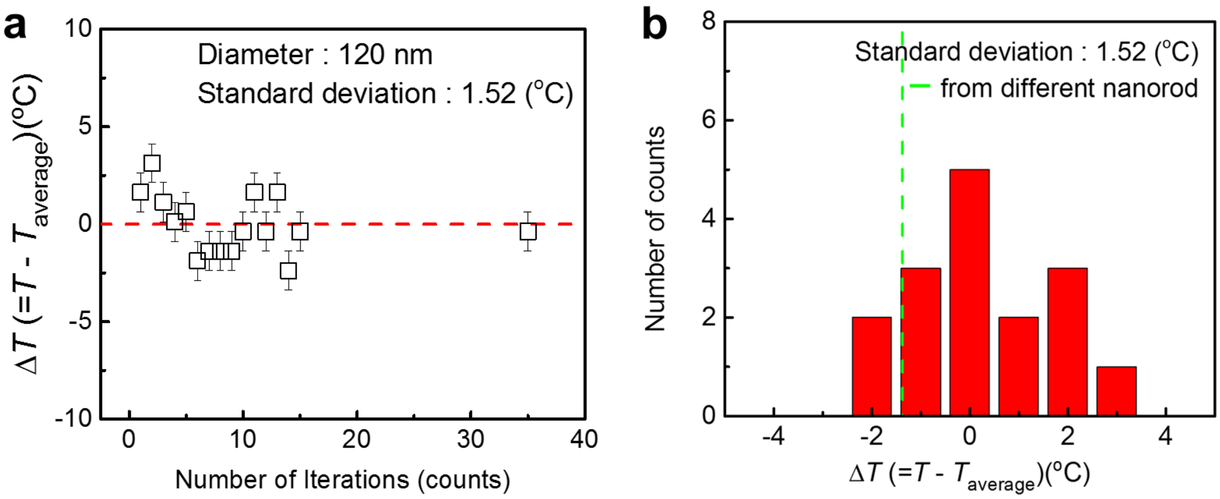

Supplementary Figure 3. Cyclic heating experiments for crystallization of a Pt-based MG nanorod with a diameter of 120 nm. **a,** The deviation (ΔT) between the crystallization onset temperature (T_c) of each cycle and the average onset temperature ($T_{average}$) is plotted with respect to the number of iterations. **b,**

The distribution of T_c's is within +/- 2 °C . In addition, after 50 + cycles, the onset temperature from a different nanorod with the same diameter (~120 nm), was measured and the temperature difference was marked (-1.7 °C, denoted with a green dashed line) from the average onset temperature of the first sample. The cyclic heating experiments show that the crystallization kinetics are not affected by factors such as possible chemical composition changes and electron beam effects.

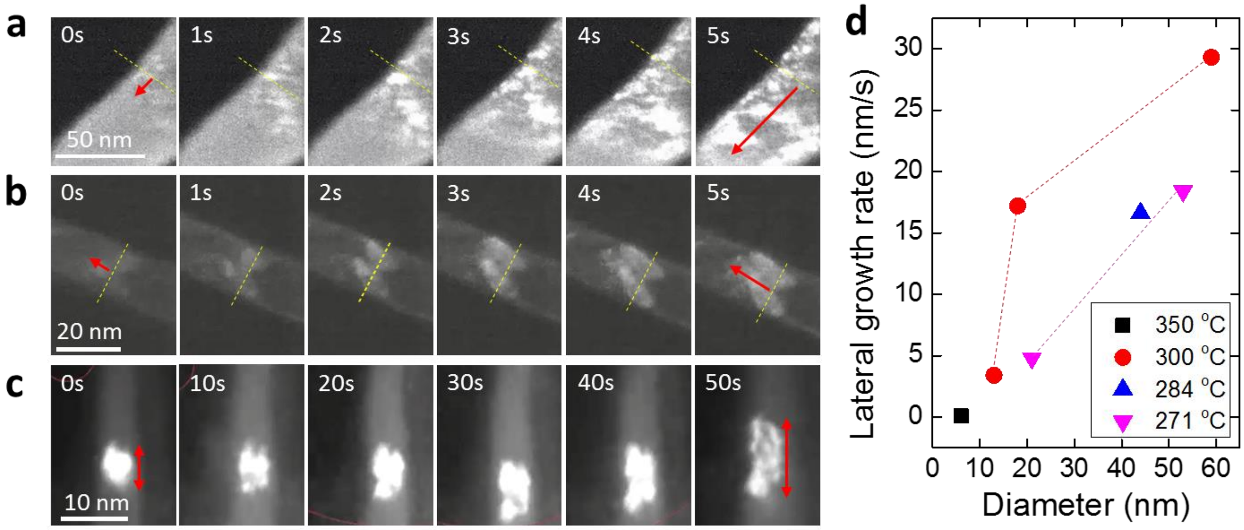

Supplementary Figure 4. Direct verification of slowed grain growth with decreasing nanorod diameter. **a-c,** *In-situ* TEM characterizations of the grain growth rates in MG nanorods of various diameters. **a, b,** Snapshot DF TEM images to compare the growth kinetics in nanorods of two different diameters. The grain growth rate is estimated by measuring the lateral expansion of crystalline grains reflected on imaging contrast change (red arrows) in the same temperature window of 271.5 °C - 273 °C. The grain growth rate for the thicker nanorod (a, ~53 nm diameter) is ~18 nm/sec, and the rate for the thinner one (b, ~21 nm diameter) is ~4 nm sec^{-1}. **c**, Snapshot DF TEM images to show the crystalline grain growth in a ~6 nm nanorod under isothermal heating at 350 °C. **d,** Correlation of grain growth rate with nanorod diameter measured at various temperatures.

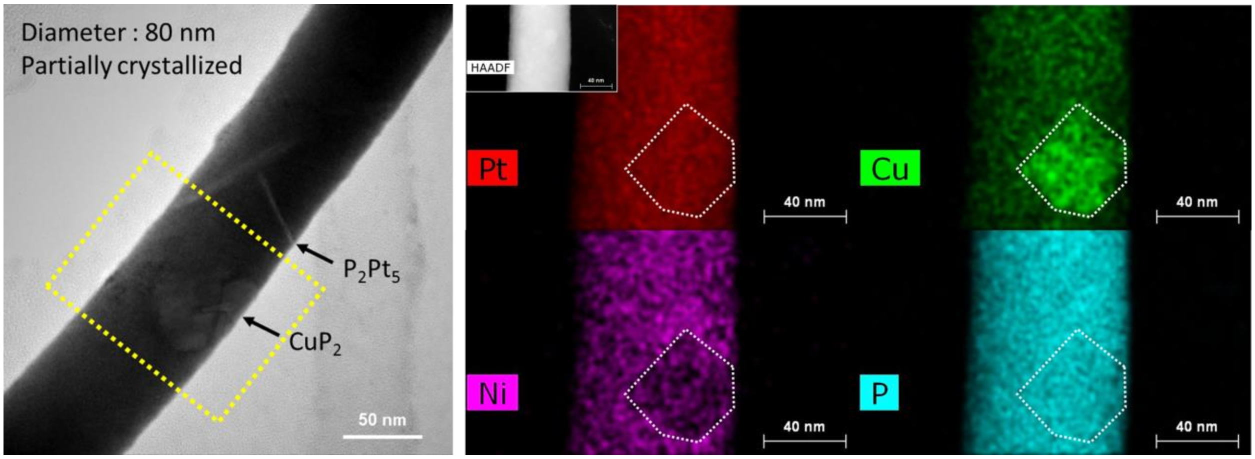

Supplementary Figure 5. A partially crystallized Pt-based MG nanorod with a diameter of 80 nm. BF TEM image (left) shows crystallized regions, which are marked with arrows. Chemical maps were acquired from the yellow boxed region and shown on the right. We observe a Cu-rich (and simultaneously Ni-poor) phase in the crystallized region, marked by the white dotted lines. Based on previous reports[1], the Cu-rich/Ni-poor phase is likely to be CuP_2. From the BF TEM image, we also observe a sharp line that is of lighter intensity. This may be a P_2Pt_5 phase, based on the previous report[1].

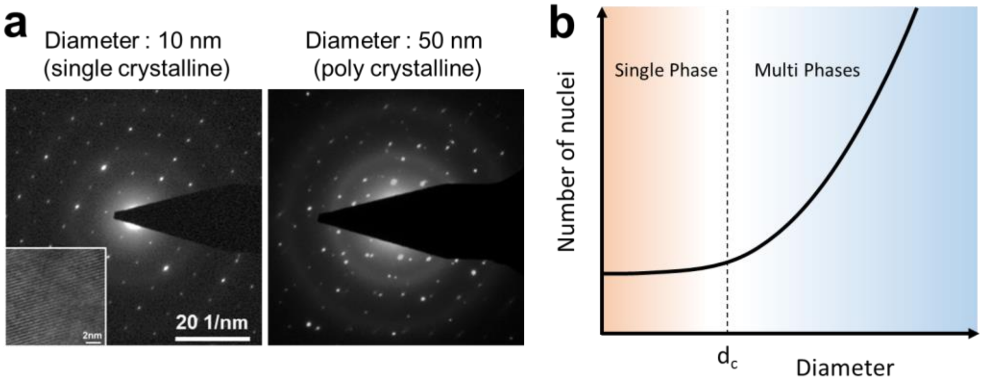

Supplementary Figure 6. Single vs. poly-crystallization. **a,** Selected area electron diffraction patterns (SAED) obtained from a 10 nm (left) and a 50 nm (right) nanorod, which shows the effect of the reduced

probability in small nanorods. **b,** Correlation of number of nuclei with nanorod diameter. Below a critical diameter, the number of nuclei present in a nanorod may be one or zero, which can result in a complete crystallization from a single nucleation.

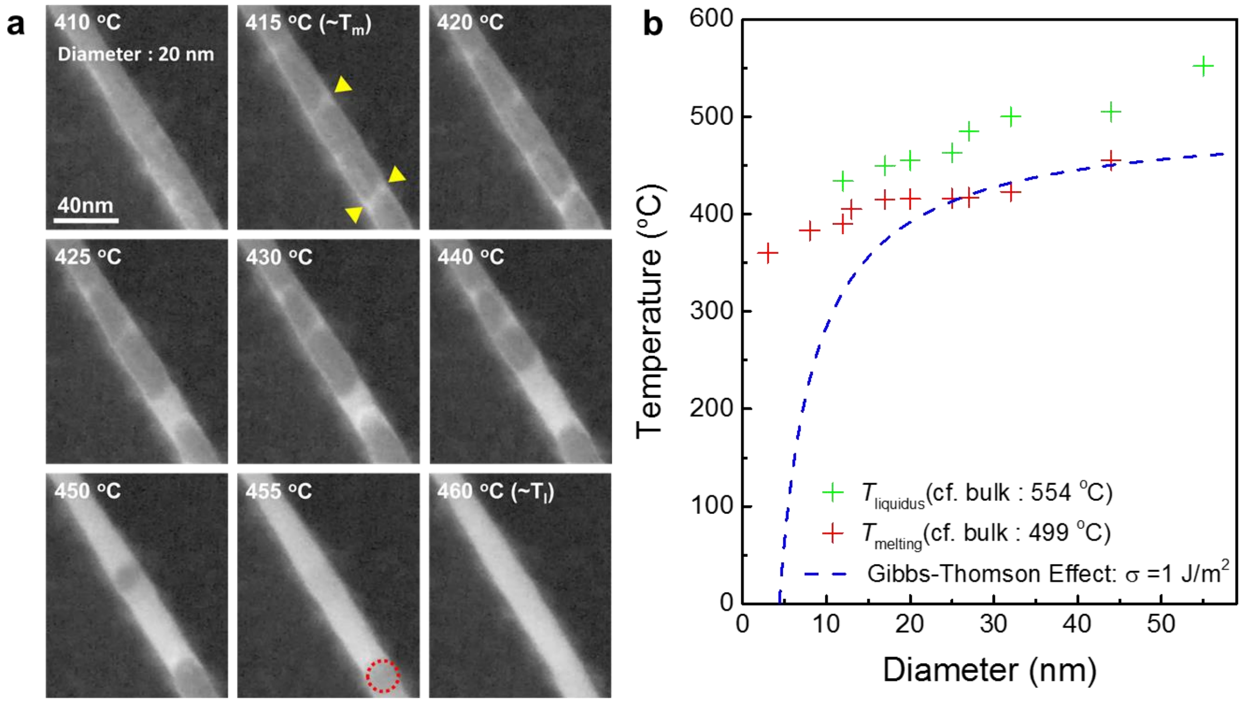

Supplementary Figure 7. Melting temperature (T_m) and liquidus temperature (T_l) of nanorods. **a,** Snapshot DF TEM images to determine the T_m and T_l. The yellow arrows (top middle) indicate where the crystal matrix started to change into the liquid phase, reflecting the T_m. The T_l was determined when the last crystalline droplet (marked by red dashed circle at the bottom middle) was completely transformed into the liquid phase (bottom right). **b,** A summary of measured diameter-dependent T_m and T_l and calculated Gibbs-Thomson effect (dotted blue line).

Supplementary Notes:

Supplementary Note 1: Cyclic heating experiments to confirm the reliability and reproducibility of T_c

Two experiments were performed to check reliability and reproducibility of our *in-situ* TEM heating experiments to measure T_c. First, we used energy dispersive X-ray spectroscopy (EDX) to check the chemical compositions of the studied nanorods before, during, and after heating inside TEM *in-situ*. We acquired the EDX spectra on three different size nanorods. No detectable changes were observed in the EDX spectra irrespective of heating conditions (Supplementary Figure 2).

Due to overlapping peaks in the EDX spectra (Phosphorous, P and Platinum, Pt, for example), the EDX analysis is not accurate enough to completely rule out any chemical effects on the crystallization kinetics. We perform repeated crystallization experiments (over 50 heating and cooling cycles) on the same nanorod to confirm the reliability of our *in-situ* heating experiments inside TEM. A large nanorod with a diameter of 120 nm was selected for cyclic heating experiments, so that the stochastic nature of reduced probability of nucleation does not play a role. The experiment procedure consists of a cyclic heating and cooling process on the nanorod with four steps; (1) heating from room temperature to 500 ^oC with a heating ramp rate of 0.67 ^oC sec^{-1}, (2) heating from 500 ^oC to 900 ^oC with a heating ramp rate of 10^6 ^oC sec^{-1}, (3) holding at 900 ^oC for less than 3 seconds and (4) cooling down from 900 ^oC to room temperature with a cooling rate of 10^6 ^oC sec^{-1}. We repeated the temperature cycle over 50 times and measured T_c from the 1st to 15th and the 36th cycle using SAED TEM movies. Supplementary Figure 3a

shows the deviations in T_c (ΔT) from the data set, which is the difference between the onset temperature of a particular cycle (T_c) and the average onset temperature ($T_{average}$). The standard deviation has been calculated using the "corrected sample standard deviation". The equation is as follows;

$$s \text{ (corrected sample standard deviation)} = \sqrt{\frac{1}{N-1}\sum_{i=1}^{N}(T_{c,i} - T_{\text{average}})} \quad (1)$$

where N is the number of experiments (or cycles). The standard deviation of the cyclic experiment is only 1.52 ^{o}C, showing that T_c can be reliably measured. In addition, after 50 + cycles, T_c from a different nanorod, which has the same diameter (~120 nm), was measured. Its T_c is different by only 1.7 ^{o}C, denoted with a green dashed line in Supplementary Figure 3b, from the average onset temperature of the first sample. These experiments reveal that the crystallization kinetics is not affected by 1) any composition changes that may be induced by *in-situ* heating or the electron beam, 2) any radiation damage due to the electron beam during the *in-situ* experiments, and 3) any carbon layer build-up due to prolonged exposure to the electron beam.

Supplementary Note 2: Growth rate difference in a nanorod with a narrow region

Figure 4 in the main text shows a series of DF TEM images from an *in-situ* TEM movie (Supplementary Video 4), which clearly displays the changes in the growth rate for the ~17 nm nanorod with a narrow region (~9 nm). Orange triangles mark the narrow region (Fig. 4a in the main text). The nanorod was heated from room temperature to 900 ^{o}C, with a constant heating ramp rate of 10 ^{o}C sec^{-1}. The grain grew from the left to the right. Once the growth front (yellow

dotted line) reached the narrow region (360 ~ 370 ^{o}C), the growth rate decreased significantly, evident by the long time the growth front spends to pass the narrow region (Fig. 4 in the main text). The growth mode appears to remain the same throughout the entire growth time, which is clear in the Supplementary Video 4. Thus, we conclude that the slowed growth front suggests increase in viscosity.

Supplementary Note 3: Direct verification of slowed grain growth with decreasing nanorod diameter

The diameter-dependent apparent viscosity should affect the grain growth rate in glassy nanorods, which can be directly measured during *in-situ* heating inside TEM. Supplementary Figure 4a, b show the snapshot DF TEM images that track grain growth in nanorods of two different diameters in the same temperature window. The grain growth rate is estimated by measuring the lateral dimension of the areas with bright intensity in the images. Grains grow ~5 times faster in the thicker (~53 nm) nanorod over the thinner (~21 nm) one. Supplementary Figure 4c shows snapshot DF TEM images of a grain growth in a ~6 nm nanorod under an isothermal condition at 350 ^{o}C. The measured growth rate is ~0.1 nm sec^{-1}, which is over two orders of magnitude smaller than those observed in Supplementary Figure 4a, b. Figure 4d shows the grain growth rate for the nanorods of various diameters, which confirm the suppressed grain growth (thus, enhanced apparent viscosity) for thinner nanorods. In addition, we note that no grain growth occurs in extremely thin nanorods below ~5 nm (Supplementary Figure 4c and Fig. 2d in the main text), which may suggest the critical nucleation size. This critical size is

determined by thermodynamic energy barriers[2,3], while the critical size (~25 nm) at which a minimum T_c occurs reflects a kinetic phenomenon.

Supplementary Note 4: Discussion of the growth mechanism

Our *in-situ* data also present an opportunity to study growth modes. We observe that for larger nanorods, multiple crystalline phases come out with chemical heterogeneity. Supplementary Figure 5 shows a partially crystallized, 80 nm nanorod. The rod was heated *in-situ* at a 0.67 ^{o}C sec^{-1} heating rate, and was quickly quenched back to room temperature when we observed partial crystallization. The EDX chemical mapping shows a Cu-rich/Ni-poor crystalline grain, which suggests a crystalline phase with a composition different from the glass composition. We think that this Cu-rich region may be a CuP_2 phase. We also observe a needle-like phase, which we think is P_2Pt_5 phase based on Legg et al[1]. The two phases are not next to each other and they do not appear to grow cooperatively. Thus, it is unlikely that the crystallization mode is eutectic. It is unclear if this is primary crystallization because of limited temporal resolution of the *in situ* TEM data.

Supplementary Note 5: Single vs. poly-crystallization

The reduced probability of nucleation in small rods suggests that a single nucleation event could cause a complete crystallization in small rods. We have indeed observed single-crystalline-like grain growth in thinner nanorods, typically below ~30 nm in diameter (Supplementary Figure 6a, left) while poly-crystalline-like growths are observed for thicker rods

(Supplementary Figure 6a, right) under the identical heating condition of a 0.67 ^{o}C sec^{-1} heating rate. This suggests a critical size below which a single nucleation leads to complete crystallization, as schematically drawn in Supplementary Figure 6b with d_c denoting the critical size. The reduced probability of nucleation in small rods helps to explain the increase of T_c.

Supplementary Note 6: Statistical studies on the crystallization onset temperature of nanorods (Fig. 5 of the main text)

A large number of nanorods with a diameter of 10 $\pm$ 1 nm, 20 $\pm$ 1 nm, 50 $\pm$ 2 nm and 120 $\pm$ 3 nm were heated from room temperature to 500 ^{o}C with a constant ramp rate of 0.67 ^{o}C sec^{-1}. The onset temperatures (T_c) of each experiment were collected from non-tapered nanorods of uniform diameters, using dark field (DF) or selected area electron diffraction pattern (SAED) TEM movies, during heating. Figure 5a shows the temperature scatter, the deviation (ΔT), which is the difference between the onset temperatures (T_c) and the average onset temperature ($T_{average}$). We observe that the scatter becomes smaller as the size of nanorods increases. To see the scatter more clearly, the standard deviation has been calculated using the corrected sample standard deviation. The calculated standard deviations of 10 nm, 20nm, 50nm and 120 nm samples are 25.5 ^{o}C, 14.6 ^{o}C, 5.5 ^{o}C and 1.5 ^{o}C, respectively, as shown in Fig. 5b in the main text. The obtained values strongly suggest the stochastic phenomenon can be seen in smaller sized samples. Thus, we can consider the nucleation event in a small nanorod (below ~30 nm) suggests the critical role that the reduced probability of nucleation plays on crystallization kinetics. We note that even in the presence of the temperature scatter, the non-monotonic behavior of T_c still holds.

Supplementary Note 7: Melting temperature (T_m) and liquidus temperature (T_l) of nanorods

The melting temperature (T_m) and the liquidus temperature (T_l) were measured as a function of the nanorod diameter. The melting temperature was determined when the crystal matrix started to change into the liquid phase (see yellow arrows in Supplementary Figure 7a) and the liquidus temperature was determined when the last crystalline droplet (marked by red dashed circle in Supplementary Figure 7a) was completely transformed into the liquid phase. We observe a decrease in the melting and liquidus temperature with decreasing nanorod diameters, as shown in Supplementary Figure 7b. This shows the Gibbs-Thomson effect. The decrease in T_m and T_l, as expected from the Gibbs-Thomson, indicates that the thermal contact between small nanorods and the *in-situ* TEM thermal chip is reliable and excellent and that the thermal conductivity of the nanorods does not change significantly for small rods. The calculated Gibbs-Thomson effect[4] (dotted line in Supplementary Figure 7b) works well down to ~ 25 nm. Interestingly, the data do not fit the universal behavior under 15 nm. The cause for this deviation is not clear yet. Some studies suggest that it may be explained by considering the particle as a cluster of atoms. As the cluster decreases in size, surface atoms are bound to the 'bulk' more loosely due to the decreasing number of bulk atoms. Consequently, the melting temperature will deviate from the 1/D curve[5]. The change in the ratio of the surface-to-bulk energy may also lead to a structural transition[5,6] and a reduction in surface tension[7,8].

Supplementary References

1 Legg, B. A., Schroers, J. & Busch, R. Thermodynamics, kinetics, and crystallization of $Pt_{57.3}Cu_{14.6}Ni_{5.3}P_{22.8}$ bulk metallic glass. *Acta Mater.* **55**, 1109-1116 (2007).

2 Bai, G. *et al.* The thickness dependence of the crystallization behavior in sandwiched amorphous $Ge_2Sb_2Te_5$ thin films. *Physica B* **406**, 4436-4439 (2011).

3 Cao, C. R. *et al.* Ultrahigh stability of atomically thin metallic glasses. *Appl. Phys. Lett.* **105**, 011909 (2014).

4 Nanda, K. K., Sahu, S. N. & Behera, S. N. Liquid-drop model for the size-dependent melting of low-dimensional systems. *Phys. Rev. A* **66**, 013208 (2002).

5 Kofman, R., Cheyssac, P., Lereah, Y. & Stella. A. Melting of clusters approaching 0D. *Eur. Phys. J. D* **9**, 441–444 (1999).

6 Koga, K., Ikeshoj, T. & Sugawara, K. I. Size- and temperature-dependent structural transitions in gold nanoparticles. *Phys. Rev. Lett.* **92**, 115507 (2004).

7 Samsonov, V. M., Bazulev, A. N. & Sdobnyakovy. N. Y. On applicability of Gibbs thermodynamics to nanoparticles. *Cent. Eur. J. Phys.* **1**, 474–484 (2003).

8 Sheng, H. W. Superheating and melting-point depression of Pb nanoparticles embedded in Al matrices. *Phil. Mag. Lett.* **73**, 179–186 (1996).
